# Modelling land cover change in the Brazilian Amazon: temporal changes in drivers and calibration issues

**DOI:** 10.1007/s10113-014-0614-z

**Published:** 2014-05-16

**Authors:** Isabel M. D. Rosa, Drew Purves, João M. B. Carreiras, Robert M. Ewers

**Affiliations:** 1 Imperial College London, Silwood Park Campus, Buckhurst Road, Ascot, SL5 7PY UK; 2 Computational Ecology and Environmental Science, Microsoft Research Cambridge, Roger Needham Building, 7 JJ Thomson Ave, Cambridge, CB3 0FB UK; 3 Tropical Research Institute (IICT), Travessa do Conde da Ribeira, 9, 1300-42 Lisbon, Portugal; 4 Forest Research Centre (CEF), School of Agriculture, University of Lisbon, Tapada da Ajuda, 1349-017 Lisbon, Portugal

**Keywords:** Model calibration, Land-cover change, Predictive models, Transition length, Temporal trends, Model performance

## Abstract

Land cover change (LCC) models are used in many studies of human impacts on the environment, but knowing how well these models predict observed changes in the landscape is a challenge. We used nearly three decades of LCC maps to run several LCC simulations to: (1) determine which parameters associated with drivers of LCC (e.g. roads) get selected for which transition (forest to deforested, regeneration to deforested or deforested to regeneration); (2) investigate how the parameter values vary through time with respect to the different activities (e.g. farming); and (3) quantify the influence of choosing a particular time period for model calibration and validation on the performance of LCC models. We found that deforestation of primary forests tends to occur along roads (included in 95 % of models) and outside protected areas (included in all models), reflecting farming establishment. Regeneration tends to occur far from roads (included in 78 % of the models) and inside protected areas (included in 38 % of the models), reflecting the processes of land abandonment. Our temporal analysis of model parameters revealed a degree of variation through time (e.g. effectiveness of protected areas rose by 73 %, *p* < 0.001), but for the majority of parameters there was no significant trend. The degree to which model predictions agreed with observed change was heavily dependent on the year used for calibration (*p* < 0.001). The next generation of LCC models may need to embed trends in parameter values to allow the processes determining LCC to change through time and exert their influence on model predictions.

## Introduction

Human induced land cover change (LCC) in the tropics is severely altering landscapes, causing the depletion of many species’ habitat (Gibson et al. 2011) and increasing the amount of carbon released to the atmosphere (Baccini et al. 2012). LCC models are used in many studies such as those that study the impact of building a new road (Soares-Filho et al. 2004) or those that aim to estimate carbon losses due to LCC (Galford et al. 2010). There are many models that predict future LCC (Rosa et al. 2013; Soares-Filho et al. 2006; Verburg et al. 2013), but knowing how well these models predict the observed changes in the landscape is still a challenge (Brown et al. 2013). Here, using a case study in the Brazilian Amazon we tackle this problem by investigating how well these models work and how reliant they are on the timing and time-scale of the data.

The modern era of Amazonian deforestation began in the 1960s and 1970s with colonisation schemes implemented by the Brazilian government, which aimed to relocate unemployed people from other parts of Brazil (Fearnside 2005). Later, with the healthy Brazilian economy growing fast and the increasing demand for agriculture products from other parts of the world, there was rapid expansion of large-scale agriculture (Nepstad et al. 2006b). In more recent years, however, the Brazilian government policies against illegal deforestation (Soares-Filho et al. 2010), market-based campaigns (Rudorff et al. 2011) and the coincident global economic crisis have reduced the rate of deforestation (INPE 2014) and altered the spatial pattern of forest clearings (Rosa et al. 2012).

**Fig. 1:**
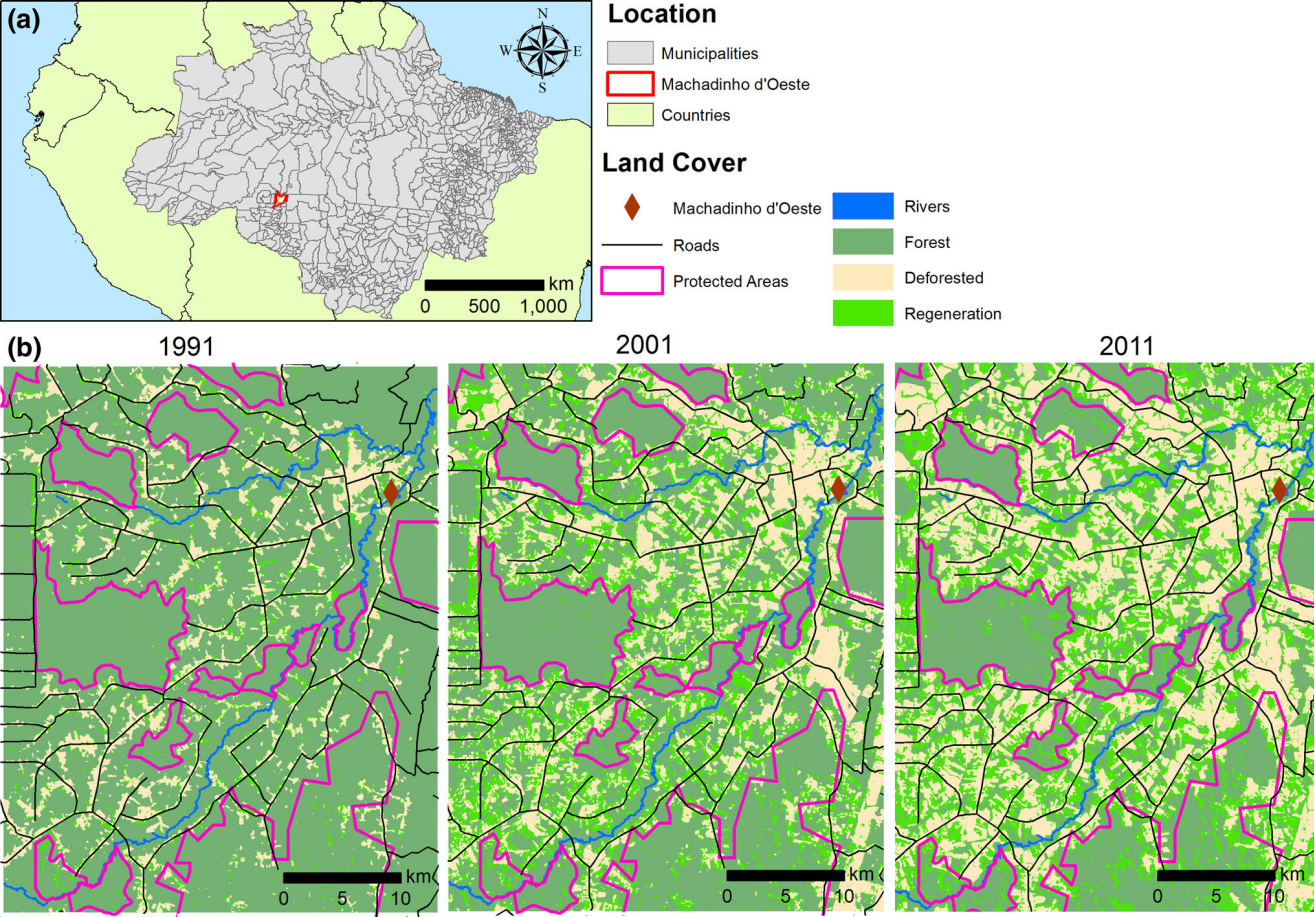
Location of study area (**a**) and land cover change from 1991 to 2011 (**b**). The maps also show the drivers of change considered in the models: settlements (Machadinho d’Oeste), rivers, roads and protected areas

Here, we focus on a study region located in the municipality of Machadinho d’Oeste, in the state of Rondônia (Fig. 1). The whole municipality shares a similar LCC history since it was all included in the planned settlement program implemented by the government in the 1980s (Batistella and Moran 2005). The majority of people in the municipality are dependent on small-scale agriculture and less than half live in urban areas (Miranda 2012). With such an intense relationship between people and agriculture it is not surprising that this municipality has been undergoing severe deforestation since the settlement was created in 1982. By 1997 it had lost 30 % of its original forest extent (Mangabeira et al. 1998), and by 2005 the original landscape had been transformed into a mosaic of remnant forest patches, secondary vegetation, pastures, agriculture lands and small urban areas (Gomes et al. 2009). The main sources of income for families in the region are livestock and coffee (Miranda et al. 2008; Gomes et al. 2009). In line with the rest of Rondônia, the livestock numbers in Machadinho d’Oeste rose sharply from 4,000 in 1989 to more than 215,000 by 2007 (IBGE 2006). Since then the rate of forest loss has declined, and in places even reversed with land abandonment having led to regenerating secondary forest.

### Land cover change models

LCC models are useful tools that can be used to provide future simulations of landscape modification under specific scenarios. These models examine and statistically define the spatial patterns of LCC in a particular time period, and use these observations to make future predictions. The choice of time period is, in most instances, limited by data availability (e.g., excessive cloud cover in satellite imagery), while sometimes it can be tied to the desire of studying the effect of a particular climate phenomena such as El Niño (Ramos da Silva et al. 2008), the effect of implementing new protected areas (Soares-Filho et al. 2006; Yanai et al. 2012) or paving a road (Soares-Filho et al. 2004). Since these models are heavily dependent on the input data used to calibrate the model, one of the main limitations of LCC models is that the parameters used in future simulations are essentially frozen in time, with the statistical description of LCC patterns estimated at a single time period and implicitly assumed to remain constant into the future. The fact that the parameters are not updated through the simulations means that the effect of a variable, such as distance to roads or the effectiveness of protected areas, for instance, remain the same through time. This is a problem because we do not know how this artefact of freezing time and assuming that the processes that drive LCC do not change propagates through model predictions to generate errors in LCC predictions.

Ideally, we would always include up-to-date parameter estimates in model simulations, but this is not feasible when predicting the future because of the self-evident lack of future data for many of the most important drivers of LCC in the tropics, such as future road network development (Ahmed et al. 2013) or future prices of agricultural products such as soybeans. As a result, models are limited in their ability to incorporate many of the constantly changing human dimensions of LCC. Modellers try to minimize this limitation by the use of scenarios (Soares-Filho et al. 2006; Maeda et al. 2011; Yanai et al. 2012), which are usually based on storylines describing suites of parameter changes.

We took advantage of a large historical database encompassing nearly three decades of LCC to run several model simulations, quantifying the degree to which freezing parameters in time can influence the model outputs. Specifically, the objectives of our study were to: (1) determine which parameters are associated with different forms of LCC (forest to deforested, regeneration to deforested or deforested to regeneration); (2) investigate how the parameter values vary through time and with respect to different processes underlying LCC change (e.g. farming, abandonment); and finally (3) quantify the influence of choosing a particular time period for model calibration and validation on the accuracy of LCC predictions. Together, our analyses are designed to examine the robustness of the modelling techniques currently used to predict LCC, with a view towards developing models that appropriately quantify the uncertainty in LCC predictions.

## Materials and methods

### Data sources and preparation

The dataset, covering an area of 1,779.80 km^2^ (37.62 × 47.31 km), is composed of land cover maps at 20 points in time during the period 1986–2011 (Carreiras et al. 2012; Prates-Clark et al. 2009). Using a high frequency time series of 30 m spatial resolution Landsat 5 Thematic Mapper (TM), the authors (Carreiras et al. 2012; Prates-Clark et al. 2009) classified these satellite images into three classes: mature forest (hereafter referred to as forest), secondary forest (regeneration) and non-forest (deforested). To model the probability of LCC in each year we used five explanatory variables, which include the most important proximate causes of deforestation in the region: location of previous deforestation (contagion) (Alves 2002; Rosa et al. 2013), distance to roads (Pfaff et al. 2007), distance to rivers (Pfaff 1999), distance to settlements (Pfaff 1999) and protected areas (Nepstad et al. 2006a). The roads (both official and unofficial) and protected areas datasets were obtained from the Instituto do Homem e Meio Ambiente da Amazônia (Imazon), and the rivers and settlements maps were obtained from the Instituto Brasileiro de Geografia e Estatística (IBGE) (Fig. 1).

**Fig. 2:**
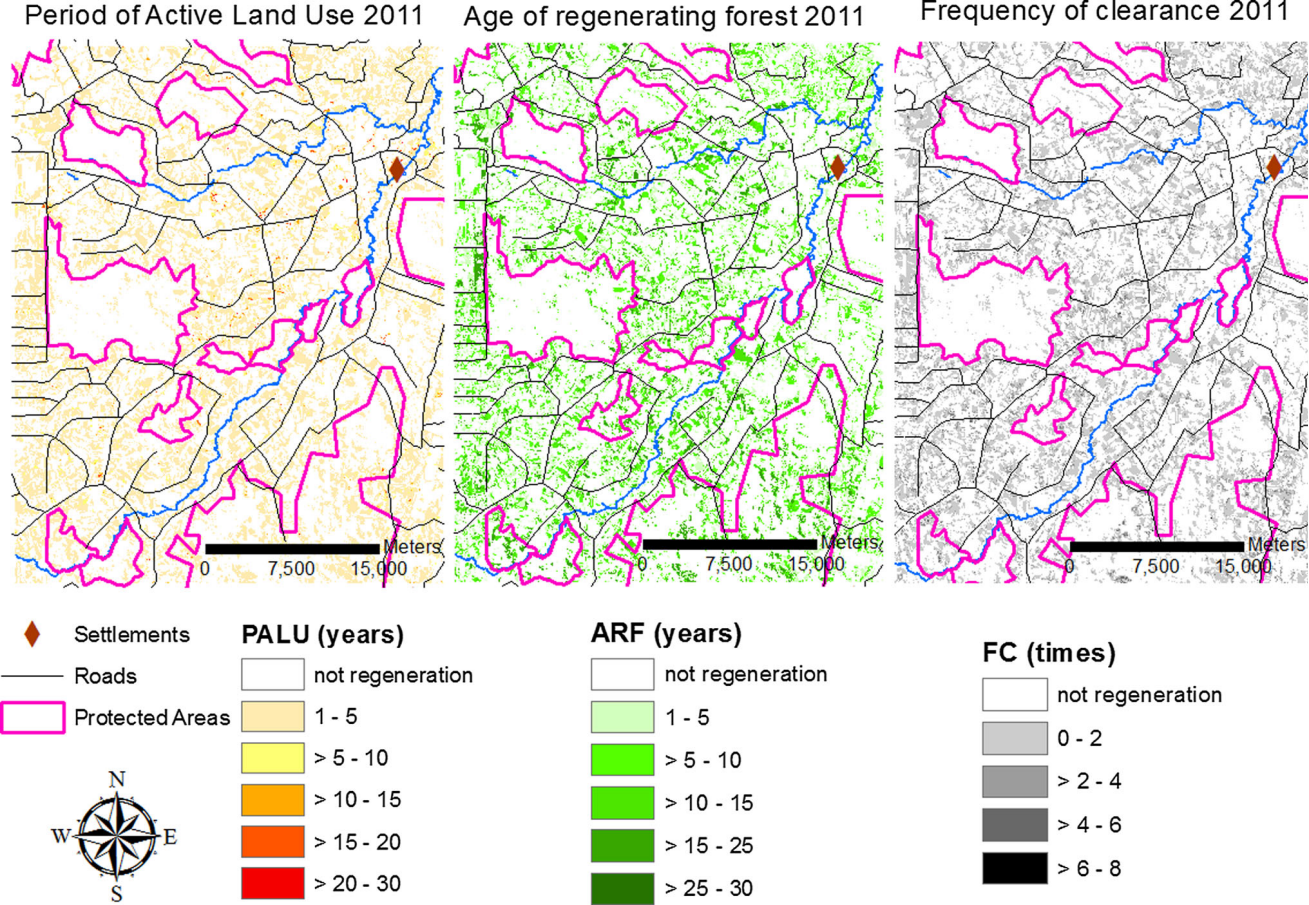
Three landscape metrics used to model the regeneration to deforested (RtoD) transition. The period of active land use (*PALU*) represents the number of years an area has been used for agriculture; the age of regenerating forest (*ARF*) is the number of years an area has been allowed to regrowth after it has been deforested; and the frequency of clearance (*FC*) is the number of times the same area has been deforested until a specific year (2011 in this example)

For areas of regeneration only, we also included three additional metrics generated from the time series of land cover itself (Fig. 2) (Carreiras et al. 2012): the period of active land use (PALU) prior to land abandonment, which is the number of years the land was used for agriculture before it was abandoned; the age of regenerating forest (ARF), which is the number of years since the land was abandoned after being deforested and allowed to regenerate; and the frequency of clearance (FC), which is the number of times a pixel of forest or regeneration was cleared (deforested) until a particular year (Carreiras et al. 2012). All data described above were converted to 30 m cell size to match the land cover dataset, using a Universal Transverse Mercator (UTM) coordinates system (zone 20 S), WGS-84 datum.

The data collected and described above were separated into two categories of variables: static (roads, rivers, settlements and protected areas) and dynamic (land cover and proportion of deforested/regeneration neighbours) variables (Soares-Filho et al. 2002). Static variables represented features that are assumed to stay constant through time (e.g. rivers) or that we lack information to be able to updated them through time (e.g. roads) and were only calculated once in the beginning of the modelling process. Dynamic variables, by contrast, represent features that change through time and were re-calculated at the beginning of each model time simulation. The main limitation of our study is our inability to add more dynamic variables in our model.

### Landscape change in the study region

We calculated the proportion of the landscape occupied by the three land cover classes at each of the 20 time points for which we had land cover maps, and determined the rate of change for each transition type in each of the 19 time periods separating adjacent time steps. We used linear regression models (R Development Core Team 2014) to test whether the rates of change in each of the three land cover transitions were constant or significantly changing through time. These analyses were performed in two ways, first using the proportion of land occupied by each land cover type as the response variable, and second using the rate of change in each land cover transition as the response.

### StocModLCC: stochastic modelling of land cover change

**Fig. 3:**
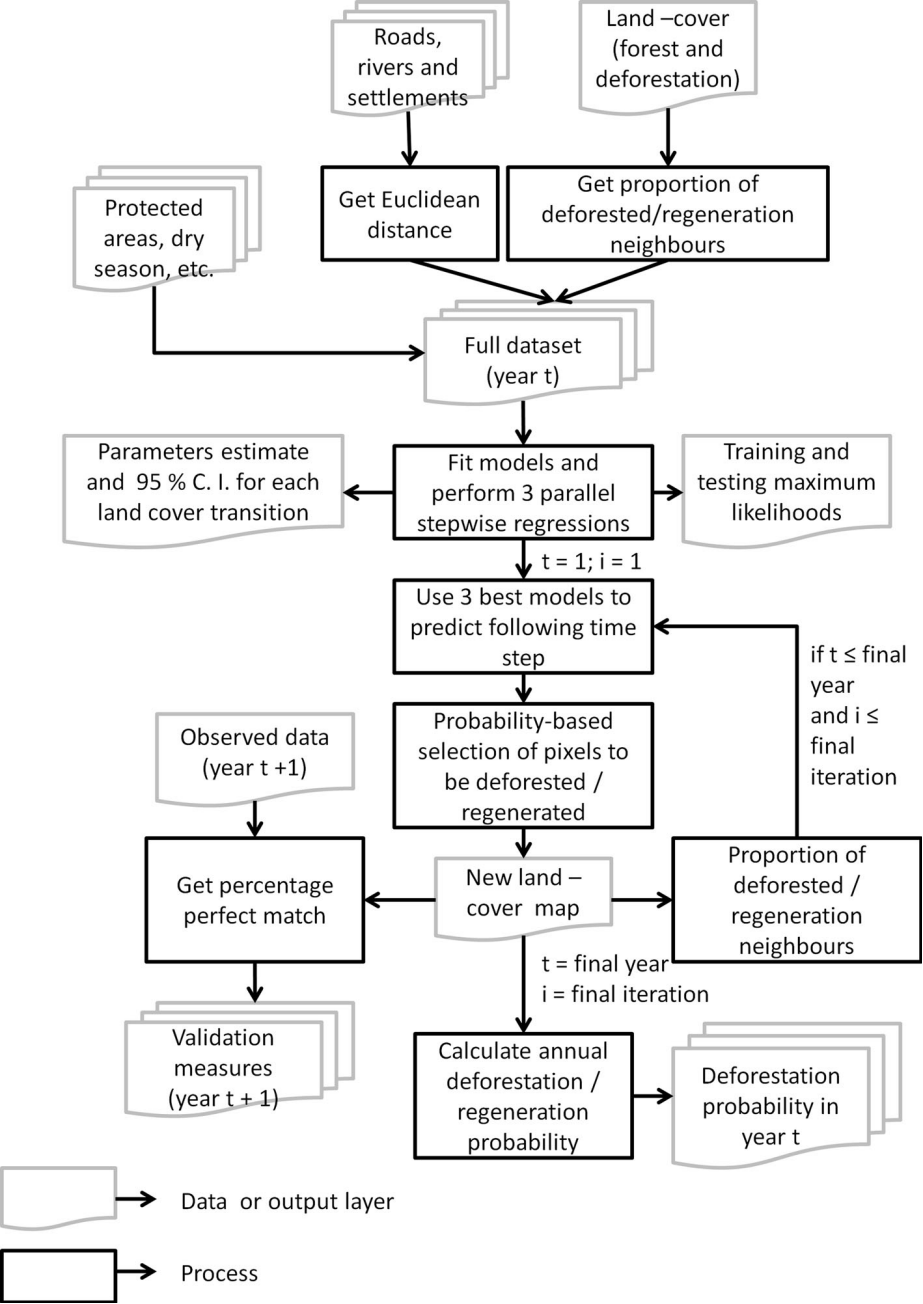
Modelling procedure flowchart. The flowchart illustrates the construction and running of the land cover change model. *i* refers to the model iteration, *t* is the first date and *t* + *n* represents the second date being modelled

Our LCC modelling approach is based on that of Rosa et al. (2013), who developed a dynamic and spatially-explicit model to predict the potential magnitude and spatial pattern of deforestation (http://stocmodlcc.net). It differs from previous models in three ways: (1) it is probabilistic rather than deterministic, allowing quantification of statistical uncertainty around the predictions; (2) the rate of LCC emerges ‘bottom up’, as the sum of local-scale probabilities driven by local processes; and (3) LCC is modelled as a contagious process, such that local rates of LCC increase through time if adjacent locations have experienced similar recent change (Fig. 3).

The fact that the dataset had three land cover classes allowed us to model three specific LCC transitions rather than the single transition of forest to deforested implemented by Rosa et al. (2013), where the model is described in full detail. Therefore, we constructed independent models of: forest to deforested (FtoD), regeneration to deforested (RtoD) and deforested to regeneration (DtoR). Given a land cover map of time *t* with three land cover classes (forest, regeneration and deforested), for each pixel of forest at time *t*, the model calculates the probability of being deforested at time *t* + *n*; for each pixel of regeneration the model calculates the probability of being deforested at time *t* + *n*; and for each deforested pixel the model calculates the probability of becoming regeneration at time *t* + *n*, with *n* being the number of years between consecutive dates.

Using as an example the transition FtoD, the model was based around *P*_*defor,x,t*_, the probability that pixel/cell *x* becomes deforested (or is converted to regeneration in the case of the DtoR transition—*P*_*reg*__*x,t*_) in a set interval of time *t*. This probability was defined as a logistic function:

1 $$P_{defor,x,t} = 1/(1 + \exp ^{ - \kappa_{x,t}})$$

such that as *κ*_*x*,*t*_ goes from minus infinity to plus infinity, *P*_*defor,x,t*_ goes from 0 to 1. Then we wrote simple linear models for *κ*_*x*,*t*_ as a function of each driver variable, or a combination of these, affecting location *x* at time *t*.

We used the C^++^ library ‘Filzbach’ (http://research.microsoft.com/en-us/projects/filzbach/) to return, for each parameter being considered in the model, a posterior probability distribution using Markov Chain Monte Carlo sampling techniques. From these distributions we extracted the posterior mean, and a credible interval, which were then used to draw parameter values, for each iteration, allowing the quantification of uncertainty around predictions.

When considering multiple drivers, a forward stepwise regression was performed in order to determine the best model for each land cover transition. The log-likelihood of each model is defined as follows:

2 $$\ell (X |s,\theta ) = \mathop \sum \limits_{x,t} log\{ Z_{x,t} P_{defor,x,t} + (1 - Z_{x,t} )(1 - P_{defor,x,t} )\}$$

where *Z*_*x*,*t*_ is the observed deforestation at location *x* at time *t*, and *s* refers to one of the models considered. At each step of the forward stepwise regression, a cross-validation was carried out by parameterising the model against a randomly selected subset of 50 % of locations (50 % of forest pixels when the transition being modelling is FtoD, 50 % of regeneration pixels when the transition being modelling is RtoD or 50 % of deforested pixels when the transition being modelling is DtoR), calculating the training likelihood, and then calculating the test likelihood on the remaining 50 % of the locations (forest, regeneration or deforested, depending on the transition being modelled).

The best models were selected as the ones with the maximum test likelihood for each land cover transition, and parameter estimates from the best models were then used to run simulations of future LCC. At each time step, after re-applying Eq. 1 we obtained an updated *P*_*defor,x,t*_ (or *P*_*reg*__*x,t*,_—probability of regeneration—depending on the land cover transition being modelled) for each location *x*, which was then deforested (or converted to regeneration) with that probability. In practice, this was implemented as follows: for each *x*, draw a random number from a uniform distribution bounded at 0 and 1, deforest (or convert to regeneration) *x* if this number is less than *P*_*defor,x,t*_ (or *P*_*reg*__*x,t*_). After these deforestation/regeneration events were implemented, *P*_*defor,x,t*_ and *P*_*reg*__*x,t*_ were calculated for every location *x* again, allowing for another round of LCC. This procedure is analogous to a weighted selection allowing for some locations with low probability to still be selected to undergo LCC, although, naturally, pixels with higher probability of change will more often change state.

Finally, given that our modelling framework has a very strong stochastic component (Rosa et al. 2013) with each individual deforestation/regeneration event being drawn using a weighted probability, each simulation was repeated for 100 iterations, using sets of parameter values randomly drawn from the posterior probability distributions. This allowed us to construct confidence intervals around our model predictions that account for the statistical uncertainty involved in parameter estimation. For each simulation, we output the predicted probability per land cover transition, the annual change on each of the land cover transitions and the new land cover map.

### Temporal variation in the causes of LCC

Our first analysis was to investigate how the set of parameters that described the impact of explanatory variables on LCC varied among LCC transition types and through time, and whether these could be associated which particular LCC processes in the region. To do this we fitted all single driver models (univariate analysis) to explain the effect of each predictor variable individually on each LCC transitions. In this analysis *κ*_*x*,*t*_ was defined by only one of the predictors at a time. After determining the parameter values associated with each driver of change in each transition and time period we used linear regression models (parameter value as the dependent variable and time/rate as independent) to test for any trend that would indicate the parameter values had changed through time (transition period) or in relation to the rate of respective LCC.

### The impact of choosing a particular time period for calibration and validation

**Fig. 4:**
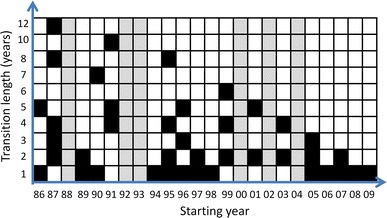
Illustration to show the combination of land cover maps and transition lengths that allow the model to predict of a land cover map for 2011 (in *black*). *Grey bars* show years with no data available

Having a large land cover dataset allowed us to test if using different calibration years (the year of the initial maps used for model calibration), different transition lengths (the number of years between the initial and final map used in model calibration), validation year (the year used for model validation), and the number of time steps the model is extrapolated into the future (number of time steps until year used for validation) had important impacts on the ability of LCC models to predict future LCC. To do this, we used all prior land cover maps to parameterise, as described above, the set of 40 models (out of a total of 66 possible models) that generated predictions for the year 2011 (Fig. 4). For example, the six-year transition period 1991–1997 models land cover in six-yearly intervals giving predictions for 1997, 2003 and 2010, but not 2011, and therefore could not be used in our analyses. By contrast, there were three time periods beginning in 1991 that did predict land cover in 2011 and were used: 1991–1995 (2011 will be the 5th time step predicted, transition length equals 4 years), 1991–1996 (4th time step predicted, transition length equals 5 years) and 1991–2001 (2nd time step predicted, transition length equals 10 years).

For each of the 40 models, the process started with a forward stepwise regression, as described above, where at each step, and for each land cover transition, models differed only in the combination of variables included in the definition of *κ*_*x,t*_ (Rosa et al. 2013). The best models were selected as the ones with the maximum test likelihood for each of the 120 stepwise regressions (40 models times three land cover transitions). These best models for each land cover transition were then used to run 40 separate simulations of LCC, each of which predicted the spatial pattern of land cover until 2011. This provided an updated *P*_*defor,x,t*_ (or *P*_*reg*__*x,t,*_) for each location *x*, which was then deforested (or converted to regeneration) with that probability.

Finally, to assess how well each of these 40 LCC models was able to predict the actual pattern of land cover we employed a pixel-by-pixel validation metric called perfect match, which tests if the model was able to predict the exact location of a land cover on that specific year. We chose this metric because, although it can be considered more rigid when compared to neighbourhood metrics such as the Kappa-family (Pontius and Millones 2011), it is more informative in showing how well the model is predicting change. Further, it does not compare our predictions to a naive or random baseline (Pontius et al. 2008; Pontius 2002; Huang et al. 2012) but with what was actually observed. First, using 2011 as an example of validation year, the observed change in land cover between the two observed maps (initial land cover and 2011) was calculated: if *x* is 1 (forest) in the initial map and 0 (deforested) in 2011, gets the value 1; or if it is 2 (regeneration) in the initial map and 0 in 2011 gets the value 2; and finally, if it is 0 in the initial map and 2 in 2011 gets the value 3. Second, we calculated the predicted change by following exactly the same procedure using the predicted maps of 2011 (the 100 land cover outputs from the 100 iterations of the model) instead of the observed map. Third, we compared the 100 predicted maps of change against the observed map of LCC. When the value of *x* (1, 2 or 3) is the same in both the observed and predicted change maps, the pixel gets the value 1, otherwise it gets the value 0. Finally, we summed all pixels with value 1 and divided this number by the total amount of observed change, including all land cover transitions. This methodology avoids validating pixels that do not change during the modelled period (Pontius Jr et al. 2004), which would inevitably lead to high but unrealistic values of perfect match. Combining the results of the 100 iterations we calculated the mean percentage of perfect match across iterations. Every land cover map available was used as validation year, which means that each model was validated against several land cover maps in time. For instance, the 2005–2006 model was validated for 5 years (2007, 2008, 2009, 2010 and 2011).

## Results

### Historical landscape change

**Fig. 5:**
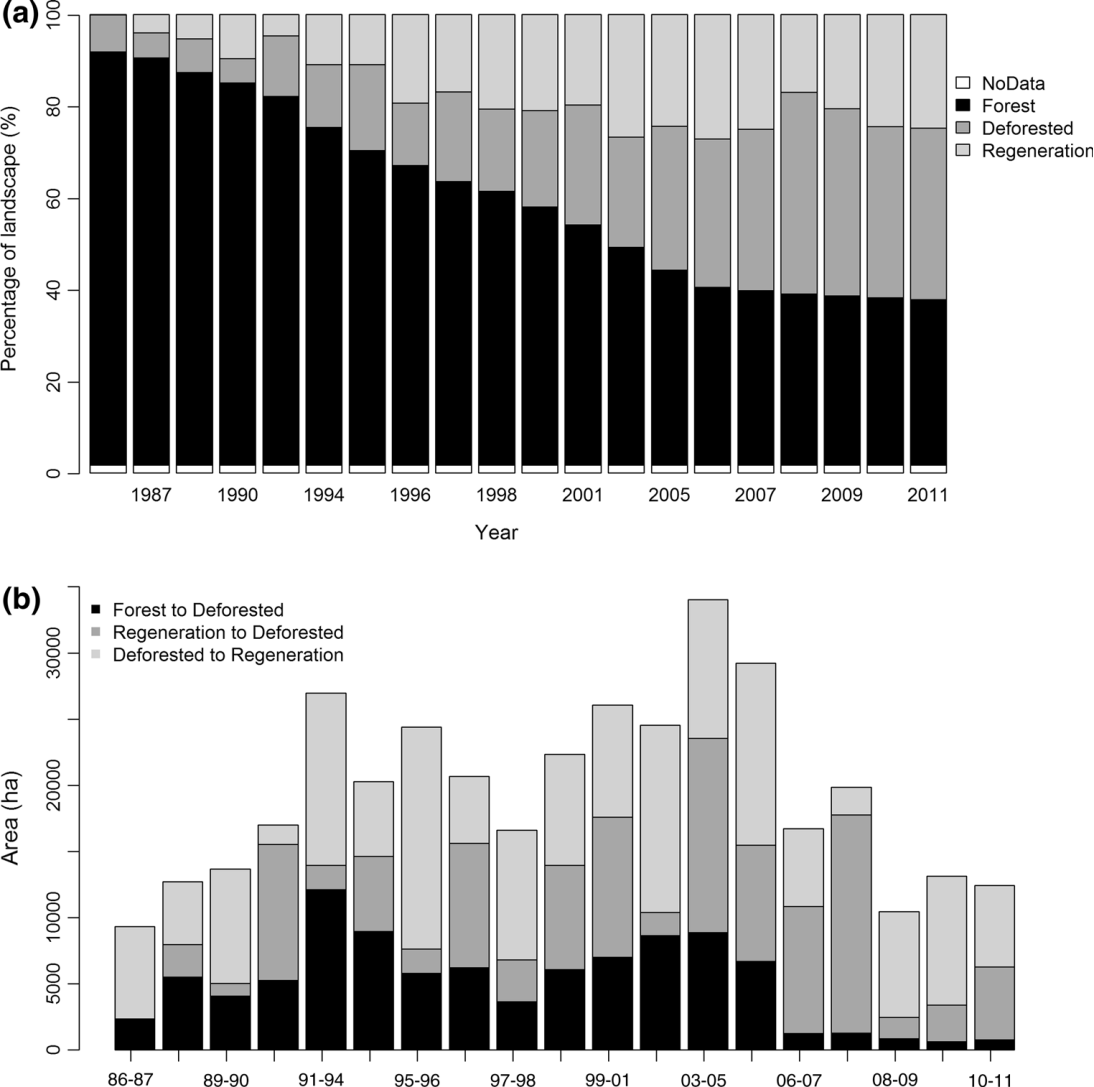
**a** Proportion of forest, regeneration and deforestation between 1986 and 2011 in study area; No Data refers to a common water mask that was applied to all the dates in the time-series. **b** The rate of change in each of the three land cover transtions: forest to deforested (FtoD), regeneration to deforested (RtoD) and deforested to regeneration (DtoR)

The landscape in our study region was highly dynamic during the period 1986–2011, with large temporal changes in the proportion of the area occupied by each of the three land cover classes (Fig. 5a). There was a strong decrease in the proportion of area occupied by forest (slope = −2.43, *t* = −36.22, *R* ^2^ = 0.99, *df* = 19, *p* < 0.001) that was accompanied by an expected increase in the proportion of area occupied by the other two classes (deforestation: slope = 1.48, *t* = 17.6, *R* ^2^ = 0.94, *df* = 19, *p* < 0.001; regeneration: slope = 0.93, *t* = 7.91, *R* ^2^ = 0.78, *df* = 19, *p* < 0.001). However, these changes appear to be stabilising with only small changes in the proportional abundance of the three land cover classes since the mid-2000s. By contrast, there was high annual variability in the rates of each LCC transition type (Fig. 5b), with no significant temporal trends detected for any transition type (|*t*| < 1.7, *R* ^2^ < 0.14, *df* = 17, *p* = 0.09).

### Temporal variation in the causes of LCC

**Fig. 6:**
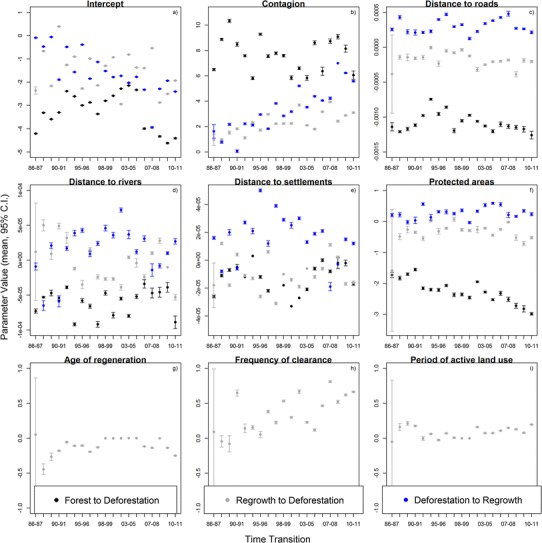
Mean values of parameters (plus 95 % CI) for all one-parameter models fitted between 1986 and 2011. In the 1986 map, the area of regeneration is very small which leads to higher uncertainties (larger CIs) in the parameters values found for this transition in this year

Parameter values found in the univariate analysis supported expected trends, with estimates varying among the three transition types (Fig. 6). Deforestation happened closer to roads and settlements but afforestation happened further away from human presence (Fig. 6c and e, respectively). Protected areas played an important role inhibiting deforestation and favouring regeneration (Fig. 6f). Furthermore, all three transitions presented some degree of contagion (Fig. 6b), although this pattern was found to be stronger in the FtoD transition than in the others.

Few parameters exhibited significant temporal trends, suggesting their effects were relatively constant through time (Fig. 6). Exceptions were the ability of protected areas to prevent deforestation, which increased in strength by 73 % through time (slope = −0.04, *t* = −7.27, *R* ^2^ = 0.76, *df* = 17, *p* < 0.001); the contagion effect of deforestation in the RtoD transition showed a strong positive trend through time (slope = 0.08, *t* = 4.54, *R* ^2^ = 0.55, *df* = 17, *p* < 0.001), varying more than six-fold, as well as the contagion effect of regeneration in the DtoR transition (slope = 0.21, *t* = 7.79, *R* ^2^ = 0.78, *df* = 17, *p* < 0.001), varying by more than three times. Finally, the effect of the frequency of clearance also revealed a significant positive trend through time in the transition RtoD (slope = 0.02, *t* = 3.48, *R* ^2^ = 0.21, *df* = 17, *p* = 0.003).

Most parameter estimates were robust to the rate of change, meaning that years with higher amounts of land changing did not result in big changes in the parameter values. The only exception was for the transition DtoR in relation to distance to settlements (there was just one settlement in the study area and two in the periphery). In years with higher rates of regeneration, it tended to happen further away from human-related activities (slope = 2.82 × 10^−10^, *t* = 4.82, *R* ^2^ = 0.58, *df* = 17, *p* < 0.001).

Parameter estimates from the 120 stepwise-based models were consistent with the results from the univariate models (Table 1). Contagion, distance to roads, and protected areas were consistently important (present in 100, 95 and 100 % of the FtoD models, and 100, 90 and 42.5 % of the RtoD models), but their effect on DtoR differ from the other two transitions. Rivers were sometimes important but with no consistent direction of effect, while settlements rarely mattered except for DtoR transition. Finally, regarding the three landscape metrics only present in the models of the transition RtoD, ARF was found to be the most regularly present on the best models (60 %), followed by FC (47.5 %).

**Table 1:** Results from the 40 modelling procedures: number of times each parameter was included in the best model (given in % of the 40 models), used for simulation on each land cover transition

	Land cover transition	Intercept	Contagion	Distance to roads	Distance to rivers	Distance to settlements	Age of regeneration	Frequency of clearance	Period of active land use	Protected areas
Included in best model (%)	FtoD	100	100	95	45	2.5	x	x	x	100
	RtoD	100	100	90	60	0	60	47.5	27.5	42.5
	DtoR	100	77.5	77.5	37.5	37.5	x	x	x	37.5
Positive percentage (%)	FtoD	5	100	0	16.67	0	x	x	x	0
	RtoD	27.5	92.5	0	54	0	0	89	64	0
	DtoR	17.5	55	100	80	93	x	x	x	87
Average	FtoD	−2.00	4.00	−0.00056	−0.00003	−0.00001	x	x	x	−1.48
	RtoD	−1.00	1.16	−0.00016	0.00001	0	−0.21	0.21	0.05	−0.36
	DtoR	−1.42	−0.65	0.00035	0.00002	0.00004	x	x	x	0.15
SE	FtoD	0.19	0.34	0.000023	0.0000043	0	x	x	x	0.07
	RtoD	0.23	0.17	0.000013	0.0000076	0	0.03	0.03	0.02	0.04
	DtoR	0.21	0.42	0.000018	0.0000067	0.0000047	x	x	x	0.06

*FtoD* forest to deforested, *RtoD* regeneration to deforested, *DtoR* deforested to regeneration; out of these, the number of times the mean of a parameter was found to be positive (given in % of the number before); average and SE of the values of the parameters in the best models for each land cover transition

### The impact of choosing a particular time period for calibration and validation

**Fig. 7:**
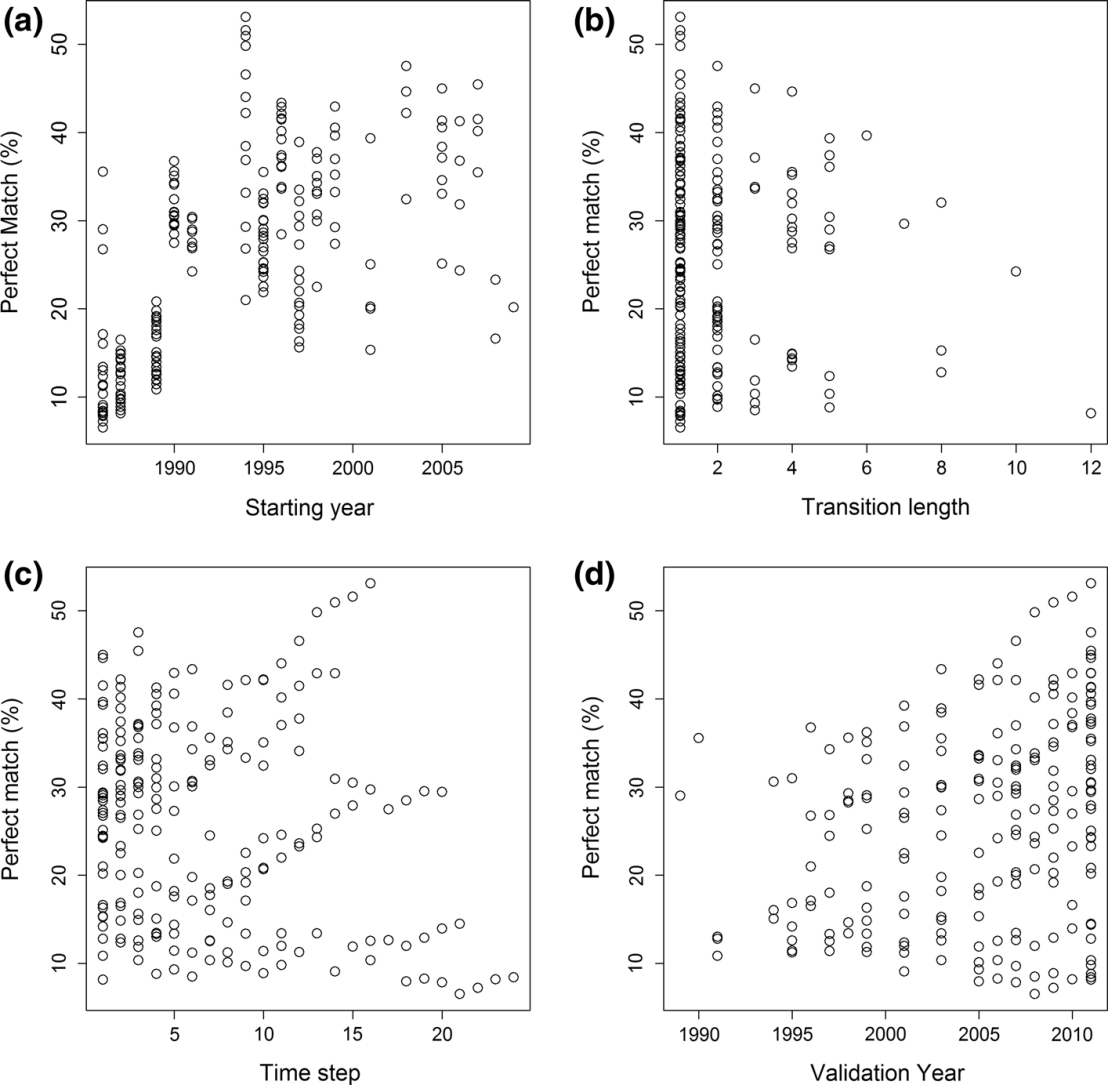
Perfect match (%) results obtained with the 40 modelling procedures by comparing land-cover change predicted and observed between model’s year of calibration and validation year (total number of validations made equals 270, more than the number of models because a single model can be validated in several years)

Calibration starting year, the number of years between calibration years (transition length), the year being validated and the time step of the model being validated had variable impacts on how well the model was able to perfectly predict the observed land cover map (Fig. 7; Table 2). We found that model performance was significantly affected by the initial year and validation year, whereas the length of the transition period and, surprisingly, the number of time steps in the future had no significant effects on their own (Table 2).

**Table 2:** Statistical analysis of perfect match results for the 40 modelling procedures, when compared to observed land cover in the year being validated, as a function of initial calibration year (Initial year), the length of the time transition being used to calibrate the model (Transition length), the model time step being validated (Time step) and finally the year being validated (Validation year)

Variable	Degrees of freedom	Sum of squares	Mean sum of squares	*F* value	*P* value
Initial Year	1	10,270	10,270	185.166	<0.001
Transition length	1	7	7	0.119	0.731
Time step	1	118	188	2.121	0.147
Validation year	1	223	223	4.018	0.046
Initial Year × Transition length	1	657	657	11.840	<0.001
Initial Year × Time step	1	4,280	4,280	77.179	<0.001
Initial Year × Validation year	1	15	15	0.264	0.608
Transition length × Validation year	1	1	1	0.022	0.882
Time step × Validation year	1	4	4	0.072	0.788
Initial year × Transition length × Time Step	1	224	224	4.036	0.046
Initial year × Transition length × Validation year	1	3	3	0.048	0.828
Initial year × Time step × Validation year	1	168	168	3.022	0.084
Transition length × Time step × Validation year	1	158	158	2.842	0.093
Initial year × Transition length × Time step × Validation year	1	14	14	0.255	0.614
Residuals	192	10,649	55		

Model predictions improved with later starting years as well as later validation years (Fig. 7a, d). Intuitively, this indicates that when trying to predict a land cover pattern it is better to use maps from a date closer to the one we are trying to predict. Furthermore, we found that using a larger transition length does not necessarily translate into more precise predictions (*p* > 0.05, Fig. 7b). However, when combined with the calibration year there was a highly significant interaction (*p* < 0.001), suggesting that once we settle on an initial year for calibration the choice of how long or short the transition length is will greatly influence our ability to predict landscape change. In particular, after choosing a recent map of land cover for modelling, a shorter transition length led to better model performance rather than a longer transition length.

## Discussion

The landscape in our study region has changed dramatically during the last 30 years, with the amount of primary forest dropping by almost two-thirds between 1986 and 2011. However, this decline in the area of primary forest has slowed down since the mid-2000s, which is probably due to a lack of good quality wood in the remaining primary forest patches that has led to a reduction in logging activity (Miranda et al. 1999), and thus a reduction in the rate at which forest is made accessible for farming. In addition, the remaining primary forest in 2011 is almost entirely sited within protected areas where logging and any other land use commonly found outside these areas such as croplands is strictly forbidden.

The analyses of the three LCC transition types can be interpreted as examining the actions of different LCC processes. Loggers, in general, are facilitators and maybe catalysts of deforestation in some settings but rarely convert forest to other uses, therefore, we associate the transition FtoD to the establishment of new farms. By contrast, well established farming activities are better represented by the transition RtoD, where there is no primary forest but regenerated forests which get routinely deforested as part of crop rotation and fallow land practices. The last transition (DtoR) can mainly be assumed to represent the process of land abandonment by farmers since no formal reforestation program has been implemented in the region.

Deforestation is still an ongoing process in the region, but the forest that is being cleared is more typically regeneration, areas that have been deforested at least once in the past. This pattern suggests that the main deforestation agents in the region are now well established farmers rather than loggers followed by new farmers (new colonists), and that the balance between regeneration and deforested land is maintained by farmers temporarily abandoning pastures and allowing regeneration to establish, before re-clearing the land in a cycle designed to maintain soil fertility in these nutrient-poor landscapes (Smith et al. 1999). The positive value found for the parameter associated with the frequency of clearance (Fig. 6h) and the fact that this value gets higher through time add support to this result by suggesting that areas that have been cleared once in the past are more likely to get cleared again.

Our study is the first to formally and statistically analyse three decades of model parameterisation associated with multiple land cover transitions in the Brazilian Amazon. Some of our results confirm well known tendencies. For example, in both transitions that lead to deforestation, forest and regeneration areas next to already deforested areas were confirmed to be more likely to be deforested in the near future (Fig. 6b), with spatial contagion being one of the most important factors in determining the rate and location of LCC (Rosa et al. 2013). However, we found that in the transition that predicts regeneration this pattern was less evident suggesting that the process of regeneration does not follow a contagious process as strongly as deforestation does, perhaps reflecting spontaneous rather than planned activities. Although this pattern will be found throughout the Amazon, where large areas of land have been deforested and later abandoned (Galford et al. 2013), there are other regions of the Amazon where well planned regeneration programs have been linked to agriculture activities such as agro-forestry (Browder et al. 2005), which might exhibit a more contagious progression of regeneration.

The role of roads in Amazon deforestation has been highlighted many times in past studies (Brandão and Souza 2006; Nepstad et al. 2001; Pfaff et al. 2007) and our study found similar results. What is interesting to note is the marked difference between land cover transitions (Fig. 6c), with the road effect being much stronger for the deforestation of primary rather than regeneration forest, and land abandonment tending to occur far from roads. In the study area there are two main roads which were built by the government as part of the settlement program in the 1980s. However, the majority of roads in the study region are unofficial roads, which are usually built by logging companies to have access to the forest to harvest the wood (Arima et al. 2005), before the area is replaced by agricultural land (Brandão and Souza 2006). These roads are then used by farmers to transport their goods to sell in the city markets. As such deforestation tend to happen closer to these roads, because farmers take advantage of these roads, but their decisions about where to deforest are also mediated by other factors such as soil fertility (Escada et al. 2005).

Protected areas have a strong preventive effect on deforestation in the Brazilian Amazon (Nepstad et al. 2006a), and we found that this effect strengthened through time as the remnant forest was progressively restricted to protected areas (Fig. 6f). Protected areas have only a limited role in preventing the deforestation of regeneration, largely because regeneration is mainly located outside of protected areas, where the great majority (90 %) of deforestation occurs. Although deforestation is illegal inside protected areas, 10 % of non-forest areas in 2011 in the study region were located inside protected areas.

Land abandonment (DtoR) tended to happen more inside protected areas than outside, which suggests that in our study area no real effort is being made to let the deforested area recover to a state closer to the original forest outside these protected areas. As such, although forest cover seems to be improving inside protected areas, these might become very isolated and their future can be undermined (Ribeiro et al. 2006). Fortunately, there are some regions of the Amazon, even within the heavy depleted state of Rondônia, where agriculture activities and forest conservation have been taking place side by side (Summers et al. 2004). As such, in these regions we would expect a higher link between regeneration and settlements and/or roads than the one we found in our study site.

The temporal analysis on model parameters revealed some degree of variation within time transitions, but for the majority of the parameters no significant trend through time. This lack of variability can partially be explained by the fact that some of these variables simply do not change through time (such as the rivers) as it does not change their relation with the land cover process being modelled. For others, such as roads, it should be expected that the estimated coefficients will have low variability with a closest fit in the time where roads and land use corresponded to each other and deviate from it as time moves away from this point in time. However, due to lack of data, we were unable to update the road map annually. This most likely affects the results found for the parameter associated with this variable.

The relatively constancy in the parameter associated with human settlements is potentially limited by the fact that there is only one settlement in our study area. As it is well known that settlements can strongly influence landscape change in the Amazon (Brandao and Souza Jr. 2006), settlements should not be assumed to be constant in the models. Their impact on the landscape is not only about the number of people that live in it, but also their distribution. Although no new settlements were created, the population of Machadinho d’Oeste followed the Amazon-wide trend of first expansion (73, 82 and 50 % raise from 1991 to 1996 in total, rural and urban population) and later concentration in towns and along roads (a contraction of 1 % in population from 2007 to 2010) (IBGE 2011). However, in the ~30 years the database covers, and given the small scale size of our study, we were unable to test for this hypothesis due to lack of spatial data on the population distribution in the region.

There were, however, some parameters that exhibited strong temporal trends, such as protected areas, which became increasingly more important in preventing deforestation of primary forests, while the landscape changes and the forest outside protected areas gets heavily degraded. Also the contagion effect of deforestation (RtoD transition) and regeneration (DtoR transition) became increasingly more important, showing how LCC strongly follows a contagion pattern. The changes found in the parameter associated with frequency of clearance show that when large areas of primary forest are unprotected, people tend to deforest those areas. However, as the landscape evolves and these forests become scarce (and constrained to protected areas), people opt to re-deforest areas that have already been cleared.

The temporal variability found in parameter estimates needs to be incorporated into models, although this represents a considerable challenge to a modelling discipline that rarely quantifies the uncertainty around their predictions (Rosa et al. 2013, 2014). For parameters that exhibit non-directional variability, this might be possible by running stochastic models that sample parameter values from the statistical distributions of parameter estimates rather than using the ‘best’ estimate alone (Rosa et al. 2013). Parameters that exhibit directional trends, however, will require more extensive time series data to allow that trend to be quantified. The next generation of LCC models will need to embed those trends within simulations in order to allow the processes determining LCC to change through time and exert their influence on model predictions.

Further, the accuracy of LCC models was heavily dependent on the year in which models were calibrated (Table 2), suggesting that a widespread reliance on single calibration time periods in the LCC modelling literature (e.g. Maeda et al. 2011; Michalski et al. 2008; Yanai et al. 2012) may be providing biased predictions of future LCC. The reason for this dependence of model accuracy on calibration year is the non-stationarity of the processes underlying LCC. We found that models calibrated in the 1980s, soon after the establishment of Machadinho d’Oeste, were almost invariably poor at predicting current LCC, whereas those calibrated from the mid-1990s and later were generally much better. This suggests that accurate predictions of future landscapes cannot be made using maps from a long time in the past as model inputs, because the processes determining LCC are dynamic and change through time. As such, parameters included in the model that predict future landscapes should allow variation which would reflect this change rather than being kept constant assuming that the same process of LCC perpetuates through time.

The calibration year was the most important factor influencing the performance of the LCC models; however, once this was controlled the time step and the transition length became important as well. This suggests that after choosing the most recent land cover maps for modelling future landscape alterations, we achieved higher values of perfect match when we validated our annual predictions against land cover maps from a date closer to the one modelled (shorter time step). The validation results tended to get worse (or more uncertain) when we validated our predictions against a map from a distant date in time (larger time step). Finally, a similar result was found for the transition length, meaning that after choosing the most recent maps of land cover for modelling, a shorter transition length led to better model performance rather than a longer transition length. This result can be counterintuitive as people usually think that a larger transition length might be best for model calibration because a short transition length might not be representative of the dynamics of land cover in the region, rather it might represent a spontaneous event. However, as we show in this study, a long transition for a long-past period will likely generate a poor model because the processes of LCC are dynamic through time, and the aspects of it being captured by a model calibrated in the past (either with a long or short transition length) will not be able to capture new processes occurring in the landscape. Our results suggest it is better to use a short transition length from a recent calibration period than it is to rely on a long transition period from the past. However, the pattern was found to be less strong than the one found for the time step and, as such, more work needs to be done in order to further analyse this relationship.

## Conclusions

LCC models are used in many studies of human impacts on the environment, but knowing how well these models predict observed changes in the landscape is still a challenge. We used nearly three decades of LCC maps to determine that parameters associated with the different drivers of change were selected differently for each land cover transition; and these transitions, which varied significantly through time, could be associated with different landscape change activities. In addition, our model validations revealed a strong importance of calibration year and validation year in determining the predictive power of the model. Furthermore, some of the parameters associated with different LCC drivers exhibited strong directional trends. As a result, we suggest that the next generation of LCC models may need to incorporate temporal variability in the parameters associated to the drivers of changes in order to allow the processes determining LCC to change through time and exert their influence on model predictions.
